# Cardiomyocyte Regeneration

**DOI:** 10.3390/cells2010067

**Published:** 2013-01-15

**Authors:** Nanako Kawaguchi, Toshio Nakanishi

**Affiliations:** Department of Pediatric Cardiology, Tokyo Women’s Medical University, Tokyo 162-8666, Japan; E-Mail: nakanishi@imcir.twmu.ac.jp

**Keywords:** heart, cardiomyocyte, stem cell, TGF-β, differentiation, regenerative medicine, c-Kit, myocardial, infarct, cardiac stem cell

## Abstract

The heart was initially believed to be a terminally differentiated organ; once the cardiomyocytes died, no recovery could be made to replace the dead cells. However, around a decade ago, the concept of cardiac stem cells (CSCs) in adult hearts was proposed. CSCs differentiate into cardiomyocytes, keeping the heart functioning. Studies have proved the existence of stem cells in the heart. These somatic stem cells have been studied for use in cardiac regeneration. Moreover, recently, induced pluripotent stem cells (iPSCs) were invented, and methodologies have now been developed to induce stable cardiomyocyte differentiation and purification of mature cardiomyocytes. A reprogramming method has also been applied to direct reprogramming using cardiac fibroblasts into cardiomyocytes. Here, we address cardiomyocyte differentiation of CSCs and iPSCs. Furthermore, we describe the potential of CSCs in regenerative biology and regenerative medicine.

## 1. Introduction

Heart failure, which is one of the major causes of death worldwide [1,2], occurs mainly due to the dysfunction of cardiomyocytes. Cardiomyocytes are terminally differentiated cells. If there are no new cells to replace the damaged cells, transplantation is the only treatment to cure heart failure. Therefore, numerous studies have been conducted that focus on stem cells that can differentiate into cardiomyocytes. Around a decade ago, cardiomyocyte division was observed after myocardial infarction [3,4]. Several papers demonstrated that cardiac stem cells (CSCs) exist in the adult heart, and efforts were made to isolate these CSCs [5,6,7,8]. The major cell surface markers used for isolation were c-Kit and multi-drug resistance protein (MDR)-1 in various vertebrates [5,9,10,11], as well as stem cell antigen (Sca)-1 [6]. Side population (SP, positive to abcge-1) was also used as a marker [12,13,14]. Moreover, the morphology of the cardiosphere has been used for isolation [7,15,16,17,18,19,20,21,22,23,24,25]. Cardiosphere-derived CSCs and c-Kit-positive CSCs were recently used in clinical trials ([26] and [27], respectively). Transplantation of these stem cells has been successful for improving myocardial infarct (MI)-induced damage in animal models. Transplantation of bone marrow (BM) stem cells (BMCs) has also been used for a long time. The major effect of BMCs is not due to stem-cell differentiation into cardiomyocytes as previously expected, but rather in progressing angiogenesis.

Here, we address the possible roles of stem cells (derived from the heart and from other organs) for heart regeneration, including regeneration of the infarcted heart area. Furthermore, we address the potential of induced pluripotent stem cell (iPSC)-derived cardiomyocytes and direct reprogramming of somatic cells into cardiomyocytes, a strategy that has been rapidly and efficiently developed in recent years.

## 2. Stem Cells Used for Cardiomyocyte Regeneration

### 2.1. BMCs

The BM is the organ supplying hematopoietic stem cells in adult vertebrates. Stem cells from the BM circulate throughout the body. The early mesoderm can induce blood cells and cardiac cells [3,28]. The stem cells isolated as c-Kit positive cells from the BM were shown to differentiate into cardiomyocytes in an ischemic heart to regenerate a damaged heart [29]. Therefore, BMCs can contribute to the supply of stem cells for organ regeneration. BMCs have been tested for clinical use, and positive effects have been demonstrated overall as reviewed previously [30,31,32]. These positive effects are due to direct cardiomyocyte differentiation or paracrine effects on angiogenesis [33]. The differentiation into cardiomyocytes was questioned. It is rather due to the fusion of the stem cells into cardiomyocytes. [34]. The paracrine effects of angiogenesis thicken the infarcted area supplying energy and oxygen through new blood, brought from the cytokines released from the stem cells [33]. Because BM is rich in c-Kit positive cells that enter the circulation, it may be possible that BM-originating c-Kit positive cells participate in the recovery of the damaged heart.

Indeed, c-Kit positive stem cells were reported to induce angiogenesis [35], and research commenced to describe the mechanism of the recovery from infarcts using BM c-Kit positive cells [36]. The inflammatory reaction against tissue damage induces mobilization of c-Kit positive stem cells [37]. The c-Kit positive stem cells need to be activated by stem cell factor (SCF), a ligand of c-Kit [38]. If the c-Kit receptor is mutated, recovery is not observed after MI [39], suggesting that c-Kit is essential for recovery.

Recently, it was reported that the origin of mesenchymal stem cells (MSCs) affects the recovery of the infarct [40]. Although the molecular signatures of the MSCs from adipose tissue, umbilical cord blood (CB), and BM are similar, BM and cluster of differentiation (CD) 105^+^ purified CB perform better during cardiac regeneration, suggesting that the environment of stem cells can affect the regeneration. 

However, the exact role of c-Kit has not yet been clarified.

#### 2.1.1. Adult CSCs

Stem cells from the hearts of various vertebrates (mouse, rat, pig, and monkey) have been isolated. The major stem cells published around 2003 are c-Kit- and Sca-1-positive cells, cardiosphere-forming cells, and SP cells. 

#### 2.1.2. c-Kit Positive Cells

c-Kit positive stem cells appear promising for regenerative medicine because they reveal all of the stemness properties of CSCs and pluripotency. c-Kit positive stem cells have been well characterized [41,42]. However, according to the paper of Tallini *et al.*, c-Kit positive cells work as cardiac progenitors until the neonatal phase but function to modify angiogenesis in adults [43]. Tallini *et al.* reported that c-Kit positive cells appeared in the cardiac region around embryonic day 14.5, peaked after 0-5 postnatal days in the atrioventricular region, and were rare in the adult rat heart. We speculate that there are different roles for high and low c-Kit positive populations, where the high-c-kit positive population might work as cardiac progenitors and the low-c-Kit population might work as MSCs [44]. We also characterized long-term cultured c-Kit positive cardiac cells to obtain “true” CSCs. The original paper described that the properties of the cells did not change during long-term culture. However, our results showed differential gene expression, for example in MSCs and cardiac lineages. Thus, although the primary isolated cells may be similar, the character of the cells can change during passages [45]. 

Another role of CSCs is enhancing cardiomyocyte survival [46]. Insulin growth factor (IGF)-1 enhances myocyte survival, inhibiting apoptosis [47] via Akt signaling [48]. From our previous paper, an *in vitro* co-culture ELISA assay suggested that IGF-1 contributed to cardiomyocyte survival because the IGF-1 antibody blocked this survival effect. However, the molecules released from the CSCs should be different from IGF-1 because IGF-1 was suggested to be induced by cardiomyocytes [49,50]. This inducer has not yet been identified [49,50]. 

### 2.2. Cardiosphere-Forming Cells

Spheres are ball-shaped aggregations of one or several types of cells and are observed with stem cells such as neural stem cells [51] and CSCs [4]. CSC spheres, called cardiospheres, have been well characterized and are used for isolation of CSCs [7,15,16,17,18,19,20,21,22,23,24,25,26]. Approximately 20% of the cells are c-Kit positive and have an effect on regenerating an ischemic heart. Andersen *et al.* opposed the existence of the cardiosphere cells and suggested that the differentiated and beating cardiomyocytes may be contaminated cardiomyocytes [52]. 

One of the c-Kit positive cardiac cells (CSC-21E) we obtained showed a strong ability to form spheres [44,53]. We were interested in the protein associated with this aggregation; therefore, we performed proteomic analysis, comparing cell extracts of substrate-attached cells and sphere-forming cells. We found that chaperone proteins were upregulated in substrate (dish)-attached cells. Interestingly, similar studies, using other kinds of stem cells such as embryonic stem cells, also suggested chaperone regulation concomitant with differentiation [53,54].

### 2.3. Other Stem Cells Relating to the Heart

Sca-1-positive CSCs differentiate into beating cardiac myocytes *in vitro* by treatment with oxytocin. Side population cells were found in the heart and were characterized. These cells can differentiate into cardiomyocytes *in vitro* and *in vivo* [6].

Wojakowski *et al.* [55] demonstrated that mononuclear cells in peripheral blood expressed CD34/chemokine receptor type 4 (CXCR4) +, CD34/CD117 +, and c-met + stem cells (human); these cells could act as progenitors, expressing early cardiac myocyte-specific markers [GATA binding protein 4 (GATA4) and myocyte-specific enhancer factor 2C (MEF2C)], skeletal muscle progenitor markers [myogenic factor 5 (Myf5), myogenic differentiation (MyoD), and myogenin], and an endothelin-specific marker (VE-cadherin). In acute MI patients, CXCR4 receives a signal from SDF-1 in the BM through chemoattraction, and this signal is spread through the blood. These stem-like cells are thought to function by repairing the damage to infarcted hearts through differentiation into cardiomyocytes. Therefore, the cytokines that affect these cells, such as vascular endothelial growth factor (VEGF)/stromal cell-derived factor (SDF)-1 [56], or the inflammatory cytokine, macrophage-colony stimulating factor (M-CSF) [57], are also suggested to be important for inducing these signals. 

Islet-1-positive cells are considered true cardiomyocyte progenitors and appear during embryogenesis [58,59]. However, it is unclear whether these cells exist in adults.

Uncommitted precursor cells (UPCs) were identified in neonatal and adult rat hearts that were octamer-binding transcription factor (Oct)-4 and stage-specific embryonic antigen (SSEA)-1 positive [60]. Oct-4 and SSEA-1 are stemness genes expressed in embryonic stem cells and iPSCs [61,62,63]. UPCs express cardiac genes such as myosin heavy chain (MHC) and smooth muscle alpha-actin *in vitro*.

Telocytes, which are similar to interstitial Cajal-like cells (ICLCs), were found by electron microscopy of the mouse myocardium [64,65], mouse endocardium [66], and epicardium of autopsied human hearts [67]. The telocytes are extremely long cells that are speculated to nurse cardiomyocytes in the adult epicardium, likely providing cardiomyocyte progenitors (CMPs). Interestingly, telocytes are c-Kit and CD34 positive [65,66]. Furthermore, round and small CSCs as well as immature cardiomyocytes (assumed to be CMPs) were observed in the vein, near the aorta [67]. Telocytes may connect CSCs, CMPs, and cardiomyocytes.

### 2.4. Left-Atrium-Derived c-Kit Positive Cells

We isolated c-Kit positive cells from various parts of the heart, including the left atrium, right atrium, left ventricle, right ventricle, septum, and apex. Cells isolated from all parts proliferated. However, c-Kit positive cells isolated from the left atrium grew better and could be cultured for a longer period. A tiny fraction of these cells was myoD and GATA4 positive and differentiated into skeletal/cardiac myocytes when grown to confluence or when grown in myocyte differentiation medium [68]. Because these cells contain stem cells that can differentiate into many types of cells and express nestin and other neural markers (as determined by microarray), we named these cells left-atrium-derived pluripotent-like cells (LA-PCs). These cells could be differentiated into both cardiac and skeletal muscle cells in a methylcellulose-based MethoCult culture medium (Stem Cell Technologies) supplemented with interleukin (IL)-3, IL-6, and SCF. IL-3 and SCF, or a combination of these two factors, have been reported to contribute to myogenesis.

### 2.5. Similarity of LA-PCs to Other Stem Cells

Carcinogenic P19 cells show similar differentiation abilities and differentiate into cardiac/skeletal myocytes and adipocytes [69]. Mesoangioblasts, which can differentiate into skeletal myocytes, vascular cells, and other mesodermal cells, also exhibit similar characteristics. Interestingly, these mesoangioblasts are derived from the dorsal aorta or dorsal vessel and express c-Kit, CD34, and tyrosine kinase receptor for VEGF (Flk-1) (VEGF-1). LA-PCs and other CSC cultured as bulk (CSC-BCs) also express these genes, as determined by microarray analysis (unpublished results). Moreover, cardiac mesoangioblasts have been isolated from human cardiac myocyte biopsies [70]. These cardiac mesoangioblasts differentiate into three different cardiac lineages.

## 3. Signal Transduction of the Differentiation Switch Using LA-PCs

### 3.1. Microarray Analysis for Identification

**Figure 1:**
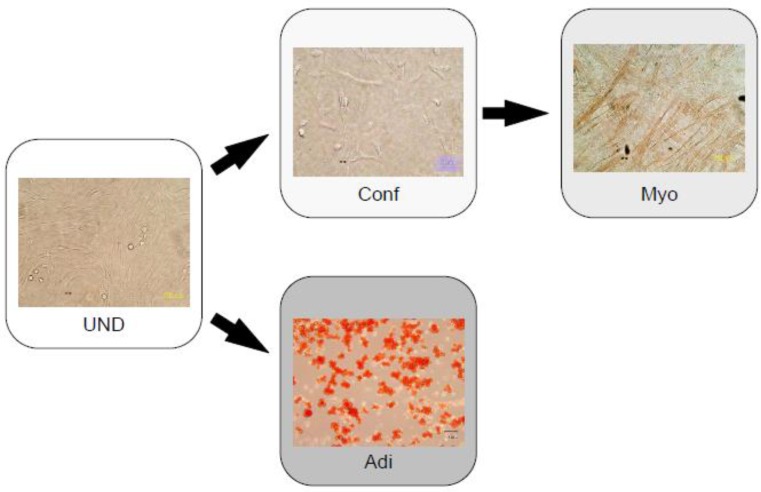
Left-atrium-derived pluripotent-like cells (LA-PCs) contain stem cells and progenitors of skeletal/cardiac myocytes (Myo) or adipocytes (Adi). Differentiated Myo was troponin I positive, and differentiated Adi was positively stained by oil red. The figure is reproduced from reference [71] with permission.

LA-PCs comprise a mixed population, consisting of undifferentiated (UND) cells and progenitors for Adi and Myo (Figure 1). We performed microarray analyses of these three cell groups (UND, Adi, and Myo) to identify the signal transduction pathway or the signal molecule responsible for the differentiation switch between Adi and Myo. We observed that the striated muscle contraction signal was upregulated in Myo, while adipogenesis and fatty acid metabolism were upregulated in Adi [71]. Therefore, we confirmed that differentiation was observed. We also demonstrated the significance of the transforming growth factor (TGF)-β pathway in signal transduction. Furthermore, we revealed that TGF-β1 simultaneously induced myogenesis and inhibited adipogenesis in a dose-dependent manner. These findings were supported by additional data. For example, TGF-R1 expression, measured by reverse transcriptase polymerase chain reaction (RT-PCR), was inhibited in Adi, consistent with the positive effect of TGF-β1 on myogenesis and the negative effect of TGF-β1 on adipogenesis. Furthermore, using RT-PCR, we demonstrated that Fst expression was upregulated in Myo but downregulated in Adi (in accordance with the microarray data). Interestingly, in previous studies, TGF-β was up-regulated 3-6 days after MI. This up-regulation *in vivo* may be associated with the myogenesis switch we observed [31]. TGF-β is expressed during early cardiac development [72]. TGF-β that enhances cardiac myogenesis of adult primitive skeletal muscle-derived cells [73] may also support the fact that the TGF-β family can work as a differentiation switch in mesodermal cell differentiation.

Our data also revealed that the expression of frizzled (fzd) is downregulated in Adi, suggesting the involvement of a Wnt signal. Crosstalk between Wnt and TGF-β is possible [72,74]. Thus, a detailed investigation of the molecules involved in these differentiation pathways is required.

In contrast, Rho GTPase signaling has been reported to be involved in the switch between adipogenesis and myogenesis [75]. Rho GTPase was downregulated in both Myo and Adi in our microarray results [44]. However, a 2-fold decrease in Myo and an 8-fold decrease in Adi were observed by both 3D-Gene analysis (Toray, Tokyo, Japan) and Agilent chip analysis (Agilent Technologies, Santa Clara, CA, USA). The magnitude of the expression can contribute to the switch.

### 3.2. Noggin and Cardiac Myocytes

We previously used a MethoCult culture to obtain small portions of beating cardiac myocytes *in vitro*. However, we failed to acquire sufficient quantities of cardiac myocytes. Therefore, we selected other TGF-β superfamily members for regulation in our microarray experiments. We observed that noggin was downregulated and confirmed this observation by RT-PCR. Interestingly, when we added noggin to the MethoCult culture, we observed a significant effect on the induction of cardiac myocytes but not on the induction of skeletal myocytes. Noggin was previously reported to act as an activator of cardiac myogenesis [76]. Bone morphogenic protein (BMP)-2 and BMP-4, play key roles in mesoderm morphogenesis. Thus, noggin, BMP, and TGF-β are important for regulating mesoderm-originated cells. Moreover, the balance and timing of the treatment can greatly alter the cell phenotype. 

Although our data are preliminary and collected using cells different from typical CSCs, our data are the first to identify the signal transduction involved in cardiomyocyte differentiation using CSCs.

## 4. iPSC-Derived Cardiomyocytes

We recently published a review article describing an *in vitro* heart disease model [77]. The differentiation of iPSCs to cardiomyocytes essentially follows the protocol of embryonic stem cell differentiation using embryonic bodies (EBs). Yang *et al.* showed that KDR(VEGFR-2)low/c-Kit negative EBs differentiate into cardiomyocyte lineages and become NK-2 transcription factor related locus 5 (NKX2.5), ISL1, T-box transcription factor (TBX5) positive but not KDRlow/c-Kit positive or KDR negative/c-Kit positive [78]. The combination of activin A, BMP4, basic fibroblast growth factor (bFGF), VEGF, and Dickkopf homolog 1 (DKK1) in a serum-free medium was necessary for cardiomyogenesis. Likewise, addition of Wnt inhibitors to BMP4 enhanced cardiomyogenesis [79]. The activin/nodal and BMP signaling pathways promote cardiac differentiation in a stage-specific manner [80]. Flk-1+ cells from EB clusters are produced in embryonic stem cell cultures without LIF, and cardiac progenitors and cardiovascular cells are differentiated from these EB clusters [81,82]. Overall, cardiomyocytes obtained from iPSCs were functionally similar to embryonic stem cell-derived cardiomyocytes [83]. iPSC-derived cardiomyocytes have similar contraction as embryonic stem cell-derived cardiomyocytes, but the contraction behavior is significantly different from cardiomyocytes derived from native tissues of individuals with comparable ages [84]. 

Ascorbic acid robustly enhances cardiomyogenesis through proliferating cardiomyocyte progenitors [85]. Ascorbic acid [86], ribosomal S6 kinase activity [87], MAPK activity [88], and small molecules [89,90,91] are known to accelerate cardiomyogenesis. However, a more concise profiling of molecular signatures is necessary to evaluate maturity and function. A unique method to purify cardiomyocytes using the large number of mitochondria within cardiomyocytes has been reported [92,93]. In this method, genetic engineering such as adding the tag is not required, and cellular damage is decreased. An alternative method was established using signal-regulatory protein alpha (SIRPA), which can select immature cardiomyocytes with fewer mitochondria [94]. 

## 5. Direct Reprogramming to Induce Cardiomyocyte Differentiation

Three transcription factors (TFs), GATA4, Mef2c, and Tbx5, are essential for cardiac myocyte differentiation for direct reprogramming of heart fibroblasts to cardiomyocyte formation *in vitro* [95]. Recently, it was shown that these TFs induce cardiomyocyte formation not only *in vitro* but also *in vivo* [96]. Furthermore, the formation of cardiomyocytes improved MI [96]. Other research groups reported that another combination of TFs (GATA4, HAND2, MEF2C, and TBX5) induces cardiomyocyte formation *in vivo*, also improving MI [97]. Although the cardiomyocyte gene expression profiles are different from the expression profiles of native cardiomyocytes, and although a retrovirus has to be used, this technique is another promising method for cardiac regeneration. If we can avoid retroviral infection and develop safer gene introduction methods, direct reprogramming is the most effective method to regenerate cardiomyocytes. 

The cell source of the direct reprogramming is the cardiac fibroblast. There are many cell types in the heart, and there are likely numerous fibroblasts that remain to be characterized. Myo-fibroblasts are induced by TGF-β, suggesting that the fibroblast cell population includes CSC-like cells and other kind of mesodermal stem cells. Characterization of the cardiac fibroblasts may lead to effective use of these cells. 

For cardiac conduction, inhibitor of differentiation (Id) 2, Tbx5, and Nkx2.5 were deemed necessary by microdissection and transcriptional profiling [98]. A combination of these TFs may induce more functional beating myocytes.

## 6. Regenerative Medicine Using Stem Cells

**Figure 2:**
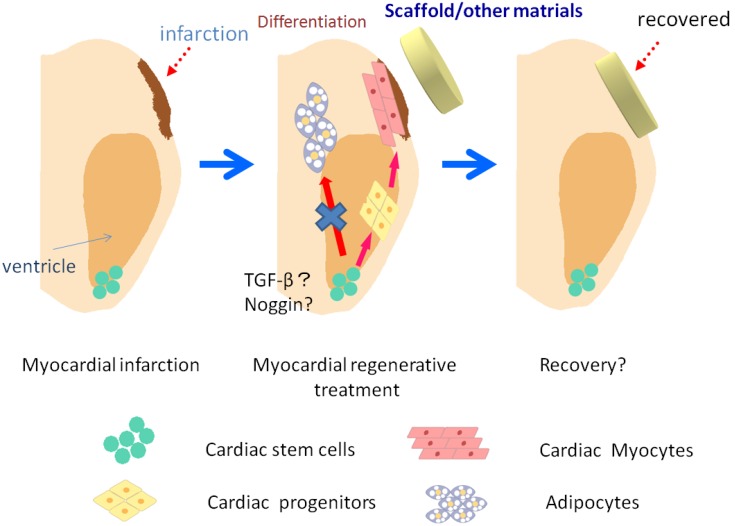
The concept of regenerative medicine. Endogenous cardiac stem cells (CSCs) are expected to differentiate into cardiac progenitors and/or cardiomyocytes, but not to other cell types such as adipocytes in the presence of cytokines, e.g., TGF-β and noggin. The CSCs can also be used as a material for transplantation with scaffold or other materials. Materials with or without CSCs that release cytokines for cardiomyocyte survival are another option.

The stem cells described above have potential application for regeneration of the heart and other organs because of the differentiation ability and paracrine effects (such as IGF-1 stimulation) of stem cells. Stem cells from adipose tissue also have potential application for cardiac recovery [99,100]. The combination of stem cells and materials, e.g., stem cells attached to collagen scaffolds, can make stem cells or CMPs/cardiomyocytes attach and settle. Implantation of the scaffold in which stem cells are embedded to the damaged tissue, to develop a cardiac tissue, represents another possibility for regenerative medicine [101]. The concept of regenerative medicine is summarized in Figure 2.Not only scaffold but also other materials have been studied for use in regenerative medicine, such as cell sheet [102,103] and synthetic polymer, hydrogel [104] and elastomers [105]. The advantage of the polymer is that the shape can be made as desired, and is easier to handle compared to scaffolds and cell sheets. However, the materials for this purpose are discussed elsewhere.

## 7. Conclusion

Resident stem cells and circulating stem cells are involved in cardiomyogenesis or angiogenesis in damaged regions of the heart, via direct differentiation or by inducing paracrine effects. These stem cells can be used for regenerative medicine. BMCs and CSCs (c-Kit positive cells and cardiosphere-forming cells) are now being employed in clinical trials. The signal transduction of cardiomyocyte differentiation is also important to guide the stem cells to cardiomyogenesis, generating a cardiac lineage and no other cell types. iPSC-derived cardiomyocytes and directly reprogrammed cardiomyocytes are important not only to replace damaged hearts, but also as they represent an *in vitro* disease model for drug discovery.
